# Low Molecular Weight Sericin Enhances the *In Vitro* of Immunological Modulation and Cell Migration

**DOI:** 10.3389/fbioe.2022.925197

**Published:** 2022-07-19

**Authors:** Juin-Hong Cherng, Shu-Jen Chang, Yaw-Kwan Chiu, Yu-Hsiang Chiu, Tong-Jing Fang, Hsiang-Cheng Chen

**Affiliations:** ^1^ Graduate Institute of Life Sciences, National Defense Medical Center, Taipei, Taiwan; ^2^ Department and Graduate Institute of Biology and Anatomy, National Defense Medical Center, Taipei, Taiwan; ^3^ Division of Rheumatology/Immunology/Allergy, Department of Medicine, Tri-Service General Hospital, National Defense Medical Center, Taipei, Taiwan; ^4^ Department of Pediatrics Songshan Branch, Tri-Service General Hospital, National Defense Medical Center, Taipei, Taiwan; ^5^ Graduate Institute of Medical Sciences, National Defense Medical Center, Taipei, Taiwan; ^6^ Department of Physiology and Biophysics, Graduate Institute of Physiology, National Defense Medical Center, Taipei, Taiwan

**Keywords:** sericin, low molecular weight, macrophage, adipose stem cell, immune response, inflammatory, cell signaling pathway

## Abstract

Sericin, a waste product of the silk textile industry, has favorable physicochemical and biological properties. In this study, we extracted a low molecular weight (MW) sericin (LMW-sericin; below 10 kDa) by a performing high-temperature and high-pressure method and confirmed the MW using matrix-assisted laser desorption ionization-time of flight and liquid chromatography–mass spectrometry. Furthermore, we determined its biological effects on macrophages and human adipose stem cells (hASCs) as cell models to investigate the biocompatibility, immunomodulation behavior, and potential signaling pathway-related wound healing *via* analyses of gene expression of focal adhesion and human cytokines and chemokines using quantitative real-time polymerase chain reaction and cytokine assay. LMW-sericin showed good biocompatibility both in macrophages and hASCs. Macrophages cultured with 0.1 mg/ml LMW-sericin displayed an improved inflammatory response shown by the upregulation of CXCL9, IL12A, BMP7, and IL10, which developed Th1 and Th2 balance. LMW-sericin also improved the differentiation of macrophages toward the M2 phenotype by significantly enhancing the expression of Arg-1, which is conducive to the repair of the inflammatory environment. Moreover, the gene expression of hASCs showed that LMW-sericin promoted the secretion of beneficial adhesion molecules that potentially activate the gene transcription of differentiation and migration in hASCs, as well as significantly enhanced the levels of PKCβ1, RhoA, and RasGFR1 as fruitful molecules in wound healing. These findings provide insights into LMW-sericin application as a potential biomaterial for wound management.

## 1 Introduction

Major biological processes such as ribosome production, stress adaptation (e.g., temperature reduction), and cell cycle control involve proteins with low molecular weights (MW) of less than 25 kDa (Müller et al., 2010). Despite their importance, smaller proteins are rather underrepresented in wound healing studies. Wound healing is a complex process of cellular and biochemical metabolisms, and necessitates a large number of nutritional substrates, particularly protein, which is essential throughout the wound-healing process. Sericin, a degumming silk protein, is composed of 18 amino acids with strong polar side groups such as hydroxyl, carboxyl, and amino groups, and is rich in serine (∼32%) and aspartic acid (∼19%) (Zhang, 2002). Unfortunately, sericin is a waste product during the manufacturing of the traditional silk textile industry and removed during the silk degumming process. As a component of small protein, sericin has not been investigated extensively. Due to its significant pH-induced instability, water solubility, and temperature, sericin alone in its pure form has a high degradability.

Sericin has attracted interest in wound dressing applications due to its excellent moisture and anti-oxidant properties, non-toxicity, non-immunogenicity, anti-tyrosinase, and pharmacological functions such as anti-coagulant and anti-cancer activities (Yang et al., 2014; Aramwit et al., 2016). Sericin can also improve the hydroxy-proline content in the stratum corneum of skin and induce fibroblast proliferation and collagen production (Tsubouchi et al., 2005; Aramwit et al., 2010). In addition, the application of sericin-based hydrogel in *in vivo* wound models significantly accelerated the wound healing process by increasing epidermal thickness and vascularization, absorbing excess exudates, and promoting cell proliferation to reconstruct damaged tissue (Ersel et al., 2016; Baptista-Silva et al., 2021). Sericin fulfills all requirements of an effective wound healing strategy and is cost-effective. Therefore, in this study, we propose sericin as a valuable protein source for wound healing.

The healing process of wounds is vastly complex with several overlapping phases including hemostasis, inflammation, repair, and remodeling (Koschwanez and Broadbent, 2011). Epithelium healing, reduction of oxidative stress, and inflammation alleviation have become critical goals to achieve alleviation of and ultimately wound healing. Although inflammatory response naturally occurs under the conditions of tissue damage or infection, the optimization strategies for shortening its stage are necessary, for example, using biomaterials to control excessive inflammation and/or release of anti-inflammatory therapeutics. One of the considered approaches is to incorporate active molecules or drugs with protein-polymer or protein composite material as main sources due to their tremendous properties. As wound healing is vital to the injured tissue, an adequate supply of protein is thus needed for consistent wound healing as they play an important role throughout the entire process (Wild et al., 2010; Yang et al., 2018). However, evidence has revealed that water-soluble proteins with a high molecular weight (MW) of approximately 10–50 kDa comprise most allergens (Hogarth-Scott, 1976; Cochrane et al., 2009). In addition, an allergen with a high MW of approximately 100 kDa is strongly associated with atopic dermatitis (Banerjee et al., 2015). Hence, the optimization of suitable low MW (LMW) proteins is essential, also, LMW protein can be absorbed rapidly and provide nutrient substrates for wound healing (Yang et al., 2018). The MW of sericin has a wide range with a maximum of 400 kDa, and peptides with MW as low as less than 10 kDa, commonly less than 5 kDa. The biological properties and MW of sericin are influenced by the extraction conditions. In this study, we aimed to obtain LMW of sericin (LMW-sericin), below 10 kDa, *via* thermal pyrolysis (with high-temperature and high-pressure (HTHP)) and diafiltration processes, which are favorable for LMW-sericin productivity (Lamoolphak et al., 2008). Several studies have shown that LMW-sericin application resulted in greater skin hydration and less irritation for the treatment of uremic pruritus in patients (Aramwit et al., 2012), and accelerated the healing process of corneal damage (Nagai et al., 2009, 2013), and protected the sciatic nerve and nerve cells related to injuries caused by diabetes mellitus (Song et al., 2013). Furthermore, Bhuyan et al. (2019) also reported that LMW-sericin may initiate some biological effects, such as pronounced anti-bacterial and anti-oxidant activities.

As studies on LMW-sericin for therapeutic in wounds remain limited, herein, we investigated the capacity of LMW-sericin treatment using macrophages and human adipose stem cells (hASCs) as cell models to determine its effects on biocompatibility, immunomodulation, and potential signaling pathways. Macrophages play dominant roles in wound healing by inducing an inflammatory response, thus classically activate M1 phenotype gradually switches toward an alternatively activated M2 phenotype, which determines the sequential inflammatory response ending in the resolution of inflammation and tissue repair (Italiani and Boraschi, 2014; Burgess et al., 2019; Li et al., 2021). Previous studies have demonstrated that the disrupted number of wound macrophages and their phenotypes caused prolonged inflammation, impaired neovascularization, fibroblast differentiation, and delayed healing (Goren et al., 2009; Mirza et al., 2009; Davies et al., 2013). In addition to macrophages, the continuous cell-cell and cell-matrix interactions, such as stem cells, that stimulate wound healing in all three overlapping phases are also essential (Ayavoo et al., 2021). For instance, Bartholomew et al. (2002) showed that mesenchymal stem cells (MSCs) had the ability to modulate immunosuppression. MSCs can cooperate with numerous immune cells (T cells, B cells, NK cells, neutrophils, and macrophages) thus regulating a balance of immune profiles and inflammatory responses (Wang et al., 2014; Nourian Dehkordi et al., 2019), and enhance wound healing and regenerate damaged tissues by increasing angiogenesis, promoting the resolution of wound inflammation, and regulating the extracellular matrix remodeling *via* paracrine interactions (Schlosser et al., 2012; Lee et al., 2016). Combined with LMW-sericin, the underlying mechanisms of stem cells in accelerating the healing process of the wound can be fully explored. Therefore, the investigation of LMW-sericin effects on macrophages and hASCs behavior is essential to determine its effects in improving the wound healing process and will provide insights into developing new therapeutic approaches for wound repair. Finally, the development of small-molecule proteins from sericin is a promising adjuvant for biological agents in wound management.

## 2 Materials and Methods

### 2.1 Isolation of Sericin Solution

The HTHP condition was used for the degumming process or separating the sericin from silk yarn (Lamoolphak et al., 2008). Using a stainless-steel pressure vessel (SS-316, AMAR, India), 16 g of cocoons of *B. mori* silkworms (Danee Silk International Company, Taiwan) were boiled with deionized water at 130°C and 2.1 Mpa for 1 h. The initial sericin extracts were then diluted with sodium chloride 0.877% (Sigma, St. Louis, MO, United States) on a Vivaflow 50 apparatus (Sartorius Stedim Biotech GmbH, Göttingen, Germany) with a partition containing a membrane with an exclusion coefficient of 10,000 MWCO PES. Next, the aqueous sericin solution was filtered and separated at a rate of 200–400 ml/min. Finally, the obtained aqueous LMW-sericin solution was frozen and freeze-dried using a lyophilizer (FD24-4S; Kingming, Taipei, Taiwan) to obtain dry sericin powder. An adjusted concentration of sericin solution was prepared by weighing and diluting the sericin powder with distilled water to an appropriate concentration (0.1, 0.5, or 1 mg/ml). In addition, the percent productivity of sericin was calculated using the equation below:

$$\mathrm{Productivity}\left(\%\right)=\frac{Silk\;weight\;before\;degumming-Silk\;weight\;after\;degumming\;}{Silk\;weight\;before\;degumming}\times 100\%.$$

### 2.2 Molecular Weight Determination

#### 2.2.1 Matrix-Assisted Laser Desorption Ionization-Time of Flight

MW was measured using a Microflex matrix-assisted laser desorption ionization-time of flight (MALDI-TOF) MS (Bruker Daltonics, Bremen, Germany). The solution was briefly centrifuged at 2,000 × g for 5 min before being spotted onto MALDI plates with sinapinic acid as a matrix. Mass spectra were recorded in positive linear mode and collected using 600 laser shots. Data were automatically acquired using Flex Analysis version 3.4 (Bruker Daltonics, Bremen, Germany).

#### 2.2.2 Liquid Chromatography–Mass Spectrometry

The sample was briefly centrifuged for liquid chromatography–mass spectrometry (LC/MS) analysis. LC/MS analysis was performed using a Q-Exactive (Thermo Scientific, Waltham, MA, United States) mass spectrometer equipped with an Accela HPLC system in Mithra Biotechnology and was controlled using the Xcalibur 3.0. Data were produced using the Protein Deconvolution software (version 4.0, Thermo Fisher Scientific, Waltham, MA, United States).

### 2.3 Fourier-Transform Infrared Spectroscopy

The structure of sericin was analyzed using a Fourier-transform infrared (FT-IR) spectrometer (Nicolet 8700, Thermo Scientific, Waltham, MA, United States) equipped with a MIRacle ™ attenuated total reflection (ATR) Ge crystal cell in reflection mode and mercury-cadmium-telluride (MCT) as the infrared detector. Briefly, the sericin solution was freeze-dried (KINGMECH, China) at −20°C under a 300 mbar vacuum for 48 h before measurement. For each measurement, 32 scans were coded with a resolution of 4 cm^−1^ and a wave number ranging from 400 to 4,000 cm^−1^. The spectra of sericin were analyzed using Origin v.9 software.

### 2.4 Preparation of Cell Culture

#### 2.4.1 Macrophage

Macrophages (U937, CRL-1593.2, ATCC, Rockville, MA, United States) were grown in Roswell Park Memorial Institute 1,640 medium (RPMI 1640, Gibco-Invitrogen, Carlsbad, CA) supplemented with 10% FBS and 18 mM sodium hydrogen carbonate. The cell density was maintained between 1 × 10^5^ and 2 × 10^6^ viable cells/ml in each T75 flask. Cells were incubated at 37°C in 5% CO_2_, and the medium was renewed twice a week.

#### 2.4.2 Macrophage Culture and PMA-Induced Differentiation

The capability of the sericin to influence polarization in the U937 cell was evaluated. U937 cells at a density of 6 × 10^6^ cells/well were incubated in a complete RPMI-1640 medium for 24 h at 37°C in 5% CO_2_. Cells were treated with 100 ng/ml phorbol 12-myristate 13-acetate (PMA; Cat. No.: HY-18739, MedChemExpress, United States) for macrophage activation, and then treated with 0.1 and 1.0 mg/ml of sericin, and 10 ng/ml of LPS (*Escherichia coli* 055: B5, Cat. No.: HY-D1056, MedChemExpress, United States), respectively. The cells were incubated for 24 h. The medium was removed before treatment with 1 μg/ml of LPS, and cells were washed with 5 ml of PBS and replenished with a complete medium. Cells treated with 10 ng/ml LPS alone were used as the control. After incubation, the cells were washed twice with PBS and resuspended in 0.5 ml of staining buffer PBS containing 1% FBS and 0.09% (w/v) sodium azide.

#### 2.4.3 Human Adipose Stem Cells

The hASCs were obtained from the adipose tissues as surgery wastes collected with informed consent from patients undergoing surgeries at the Tri-Service General Hospital in Taipei, Taiwan, with the approval of the Institutional Review Board of Tri-Service General Hospital (IRB approval number: A202105148). Approximately 1–3 ml of adipose tissues were purified as previously described (Cherng et al., 2014). The tissues were cut into small pieces and digested with a transfer solution containing 0.1 M PBS, 1% penicillin/streptomycin (Merck, Darmstadt, Germany), and 0.1% glucose (Ferak, Berlin, Germany). The sediment was then incubated in Dulbecco’s modified eagle medium (DMEM; Gibco-Invitrogen, Carlsbad, CA, United States) with 0.1% collagenase at 37°C in 5% CO_2_ for 1 day. Cells were collected *via* centrifugation at 500 × g for 5 min. The resulting pellet was maintained in keratinocyte serum-free medium (KSFM; Gibco, Carlsbad, CA, United States) containing 5% FBS, antioxidants, N-acetyl-cysteine, L-ascorbic acid-2-phosphate (Sigma, St. Louis, MO, United States), 1% antibiotic/antimycotic, and insulin. The cells were passaged every 3 days.

### 2.5 Cell Viability

Briefly, the sericin solution with the concentration of 0.1, 0.5, and 1 mg/ml was added to a well plate, respectively, and dried for 3–5 h. After drying, cells at an initial concentration of 5 × 10^4^ cells/well were seeded into a well plate in Eagle’s minimal essential medium (EMEM; Gibco, Carlsbad, CA, United States) containing 10% FBS. Cells incubated without sericin solution and with DMSO served as negative and positive controls, respectively. The culture medium was replaced every 2 days during incubation. After 24 and 72 h incubation, the culture medium was removed and a 3-(4,5-dimethylthiazol-2-yl)-2,5-diphenyltetrazolium bromide (MTT) solution at a concentration of 5 mg/ml (Sigma, St. Louis, MO, United States) was added to the culture well and incubated for 2 h at 37°C. The insoluble formazan formed in each well was then extracted with dimethyl sulfoxide (DMSO; Sigma, St. Louis, MO, United States). After shaking the plate gently for 10 min, the absorbance was measured at 570 nm wavelength using a multi-functional microplate reader (Bio-Tek ELX-800; BioTek, VT, United States). The magnitude of the optical density was used as an expression of cell viability. The tests were performed in triplicate.

### 2.6 Cell Migration

Cell migration test was performed using the QCM™ 24-well colorimetric cell migration assay (Merck, Darmstadt, Germany). First, hASCs at a density of 5 × 10^5^ cells/ml were added to the insert chambers of 24-well cell migration plate assembly. The lower chambers were filled with different stimulators including KSFM, medium contained 0.1 mg/ml LMW-sericin, medium contained 1.0 mg/ml LMW-sericin, and medium contained 10% FBS, respectively, followed by plates incubation for 24 h at 37°C. After removing the medium from the upper chambers, the inserts were transferred to a clean well containing 400 μl of cell stain solution and kept for 30 min at room temperature. The inserts were then washed, air dried, and observed through a microscope. Furthermore, the inserts were placed in a clean well containing 200 μl of extraction buffer for the next 15 min. Finally, 100 μl of the dye mixture was transferred to a 96-well plate and measured at wavelengths of 560 nm using the microplate reader.

### 2.7 Flow Cytometry

The treated macrophages were firstly resuspended with PBS for 5 min and fixed with alcohol acetic acid solution (95% alcohol + 5% acetic acid) for 5 min. The cells were then washed with PBS and pressed with 0.05% NP-40 (diluted with PBS) for 10 min. After being washed with PBS, 2% FBS was added to block the cells for 10 min; cells were then incubated with primary antibodies inducible nitric oxide synthase (iNOS; 1:100 dilution; Santa Cruz Biotechnology, Dallas, TX, United States) and arginase 1 (Arg-1; 1:200 dilution; Santa Cruz Biotechnology, Dallas, TX, United States) for 2 h at 4°C. Anti-rabbit FITC and anti-goat-rhodamine were used as the fluorescence antibodies. After being washed with PBS, the cells were fixed with 1% paraformaldehyde and analyzed *via* flow cytometer (BD FACSCalibur, BD Biosciences, New Jersey).

### 2.8 Immunofluorescence Staining

The treated cells were firstly washed thrice with PBS and fixed in 4% paraformaldehyde (Sigma-Aldrich, St Louis, MO, United States). The cells were then incubated with 0.2% Triton-X for 30 min, followed by three washes with PBS. 10% normal goat serum (Vector Laboratories Ltd., Burlingame, CA, United States) was added to block the cells, and the cells were then incubated with primary antibodies namely iNOS (1:100 dilution; Santa Cruz Biotechnology, Dallas, TX, United States), Arg-1 (1:200 dilution; Santa Cruz Biotechnology, Dallas, TX, United States), protein kinase C beta 1 (PKCβ1; 1:50 dilution; Bioss, Beijing, China), ras homolog family member A (RhoA; 1:50 dilution; Santa Cruz Biotechnology, Dallas, TX, United States), and ras protein-specific guanine nucleotide-releasing factor 1 (RasGRF1; 1:50 dilution; Santa Cruz Biotechnology, Dallas, TX, United States) at room temperature for 2 h. The secondary antibodies are anti-rabbit FITC (1:1,000; Jackson ImmunoResearch, West Grove, PA, United States) and anti-mouse-Rhodamine (1:1,000; AnaSpec, Fremont, CA, United States) were used to visualize. Cell nuclei were counterstained with DAPI. Fluorescent images were collected with an inverted fluorescent microscope (Axio Lab.A1; Carl Zeiss AG, Oberkochen, Germany) fitted with a camera (Zeiss AxioCam ICm1; Carl Zeiss AG). Semi-quantitative analysis of stained cells was carried out using ZEN 2.6 lite blue software.

### 2.9 RNA Isolation and cDNA Synthesis

Total RNA isolation was performed using TRIzol reagent (Gibco, Carlsbad, CA, United States) according to the manufacturer’s instructions (Direct-zol™ RNA Miniprep Kit, Zymo Research). RNA concentration was determined using UV-Vis spectrophotometry (NanoDrop 2000; Thermo Fisher Scientific, Waltham, MA, United States). The quality of RNA was determined *via* RNA electrophoretic separation in TBE agarose gels. cDNA was synthesized from 1 μg of total RNA using the RT^2^ First Strand Kit (Qiagen, Hilden, Germany). Briefly, samples were treated with Dnase (Qiagen, Hilden, Germany) and incubated at 42°C for 5 min. Reverse-transcription mix was added and samples were incubated at 42°C for 15 min followed by 5 min incubation at 95°C. cDNA was stored at −20°C until ready for qPCR.

### 2.10 Quantitative Real-Time Polymerase Chain Reaction

RT^2^ Profiler PCR Array System (Qiagen, Hilden, Germany) was used according to the supplier’s instructions to evaluate the expression of 84 genes of both macrophage cell line and hASCs for cytokine profiling (Cat. No. PAHS-150Z; Qiagen, Hilden, Germany) and human focal adhesions profiling (Cat. No. PAHS-145Z; Qiagen, Hilden, Germany), respectively. cDNA was thawed and added to the SYBR Green Master Mix. Real-time qPCR was amplified and detected on a LightCycler 480 Instrument II (Roche Diagnostics GmbH, Germany) using the following cycles: 1 cycle at 95°C for 10 min, 45 cycles at 95°C for 15 s, and 1 min at 60°C. A dissociation (melting) curve was also performed at 95°C for 1 min and 65°C for 2 min; readings were taken at 65°C–95°C at 2°C/min intervals. The ΔΔCt method was used to determine the relative quantity of gene expression. Microarray data were normalized against reference genes and sericin-treated approaches were compared to control-treated approaches by calculating the ΔΔCt for each gene of interest. The data analysis web portal calculates fold change/regulation using ΔΔCt method, in which delta Ct is calculated between the gene of interest (GOI) and an average of reference genes (HKG), followed by ΔΔCt calculations [delta CT (Test Group)-delta CT (Control Group)]. Fold-change is then calculated using $2^{-\text{ΔΔCt}}$ formula. In this experiment, each group and sample test had been performed in three replicates, and *p* values are calculated based on a Student’s *t*-test of the replicate $2^{-\text{ΔΔCt}}$ for each gene in the control group and treatment groups. Finally, the data were visualized as a heatmap. In addition, for the levels of PKCβ1, RhoA, and RasGFR1 analysis, the primer sequences were listed as follows (5′-3′): PKCβ1 (F: GAG​GGA​CAC​ATC​AAG​ATT​GCC​G; R: CAC​CAA​TCC​ACG​GAC​TTC​CCA​T); RhoA (F: TCT​GTC​CCA​ACG​TGC​CCA​TCA​T; R: CTG​CCT​TCT​TCA​GGT​TTC​ACC​G); RasGFR1 (F: AGG​TCA​CTG​TGC​CGC​AGA​TGA​T; R: CAG​CAC​TTT​GCA​GGA​GTT​CAT​GG).

### 2.11 Statistical Analysis

Statistical analyses were conducted using the Statistical Package for the Social Sciences version 18 (SPSS, Chicago, IL, United States). All values were expressed as mean ± SE. Analysis to determine differences in the experimental and control groups was done by one-way analysis of variance for all quantitative data. Bonferroni correction multiple-comparison post hoc tests were applied for multiple comparisons between the groups. Differences between groups were indicated by an asterisk, representing significant values (*p < 0.05, **p < 0.01, ***p < 0.001).

## 3 Results

### 3.1 Determination of Molecular Weight of Sericin

**FIGURE 1:**
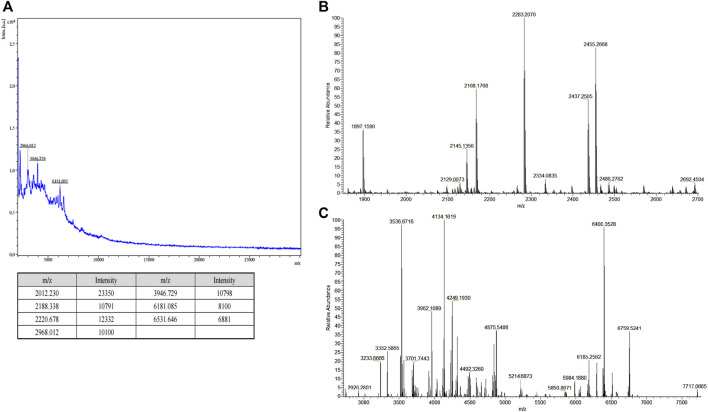
Molecular weight (MW) analysis of filtered sericin solution. **(A)** Matrix-assisted laser desorption ionization-time of flight (MALDI-TOF) mass spectra of low MW sericin solution. [*z* = 1; m/z = m (Da)]; **(B,C)** liquid chromatography–mass spectrometry (LC-MS) of low MW sericin solution at major peak result and minor peak result, respectively. [*z* = 1; m/z = m (Da)].

The sericin solution was obtained *via* thermal pyrolysis using the HTHP degumming method, resulting in a sericin with a 28.5% extraction yield. Furthermore, we conducted a diafiltration to separate the LMW-sericin and the high MW sericin (HMW-sericin). As shown in Figure 1A, MALDI-TOF displayed the mass spectra with several peaks corresponding to LMW-sericin, showing the MW ranging from 2 to 6 kDa. Moreover, as examined using LC-MS, the MW of LMW-sericin ranges from 2 to 7 kDa (Figures 1B,C). The collected LMW-sericin solution was further used the throughout experiments of the study.

### 3.2 Structure Analysis of Low Molecular Weight Sericin

**FIGURE 2:**
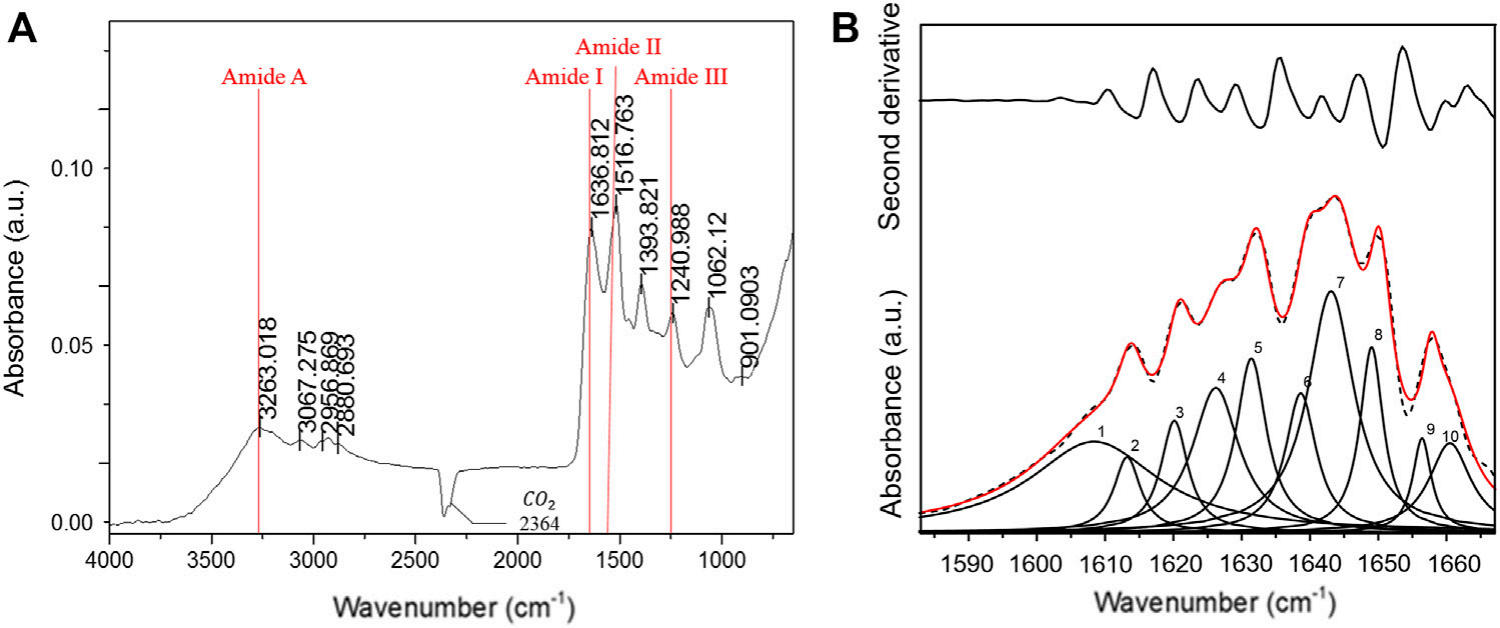
Fourier-transform infrared (FT-IR) spectroscopy low molecular weight of sericin solution. **(A)** IR spectra; **(B)** second-derivative and curve-fitted spectra, illustrated at the upper and the bottom of the figure, respectively. Ten Lorentzian curves (red solid line) were de-convoluted from the amide I band (dash line).

FT-IR spectroscopy was performed to examine the structure of the Low Molecular Weight Sericin (LMW-sericin) extracted from *B. mori* cocoons. The protein conformation was determined by identifying the peak positions of amides A, I, II, and III corresponding to CO, N-H, and C-N stretching, respectively. As shown in Figure 2A, sequentially, the sericin exhibited the characteristics of amide absorption bands of protein, such as amide A, I, II, and III specifically at 3,263, 1,637, 1,517, and 1,241 cm^−1^. Each amide absorption represents the type of molecular motion and secondary structure of the protein. Furthermore, the following secondary structure analysis was identified in the amide I region. The bands that appeared at 1,630, 1,659, and 1,644 cm^−1^ were assigned to the β-sheet, α-helix, and random coil, respectively (Figure 2B). The percentage of each secondary structure itself was estimated from the peak areas of the component bands: 27.5% for aggregated strands, 26.5% for β-sheets, 40.9% for random coils, and 5.1% for α-helices (Table 1).

**TABLE 1:** Assignment of individual amide I components.

Frequency ( $cm^{-1}$ )	Assignment	Contribution (%)
1,609, 1,613, and 1,620	Aggregated strands	27.5
1,627 and 1,632	β-sheet	26.5
1,639, 1,644, and 1,650	Random coil	40.9
1,657 and 1,660	α-helix	5.1

### 3.3 The Effects of Low Molecular Weight Sericin Treatment on Macrophage Behavior

**FIGURE 3:**
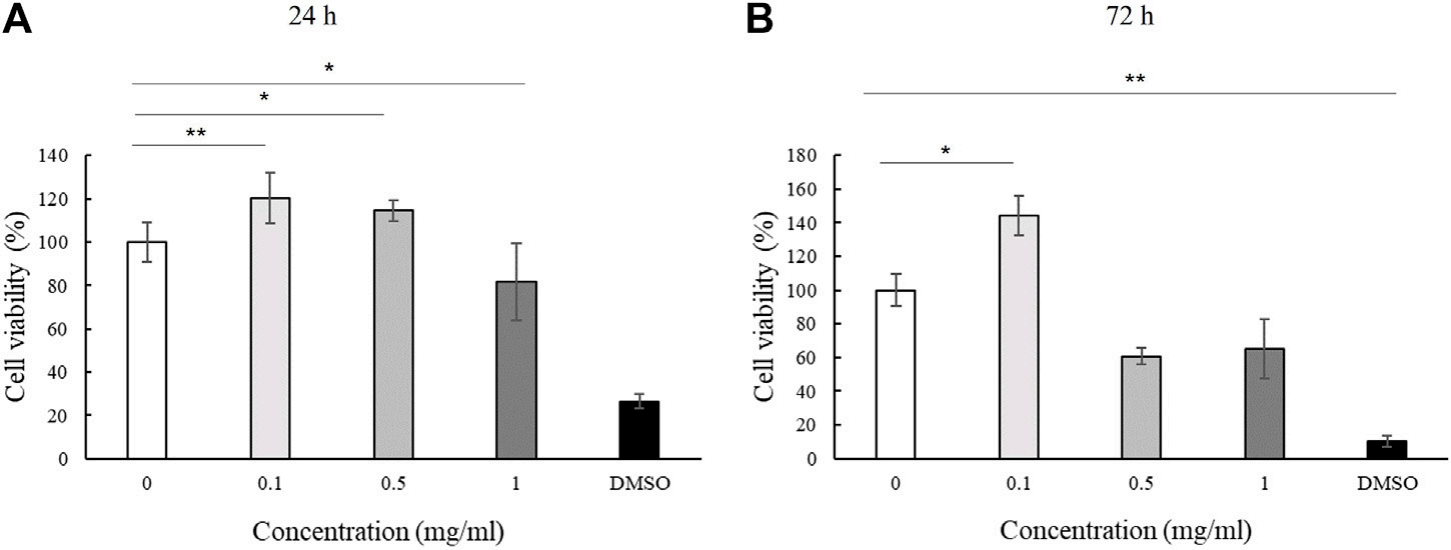
Cell viability of macrophage cultured with low molecular weight of sericin solution. (*n* = 3; *p < 0.05, **p < 0.01).

**FIGURE 4:**
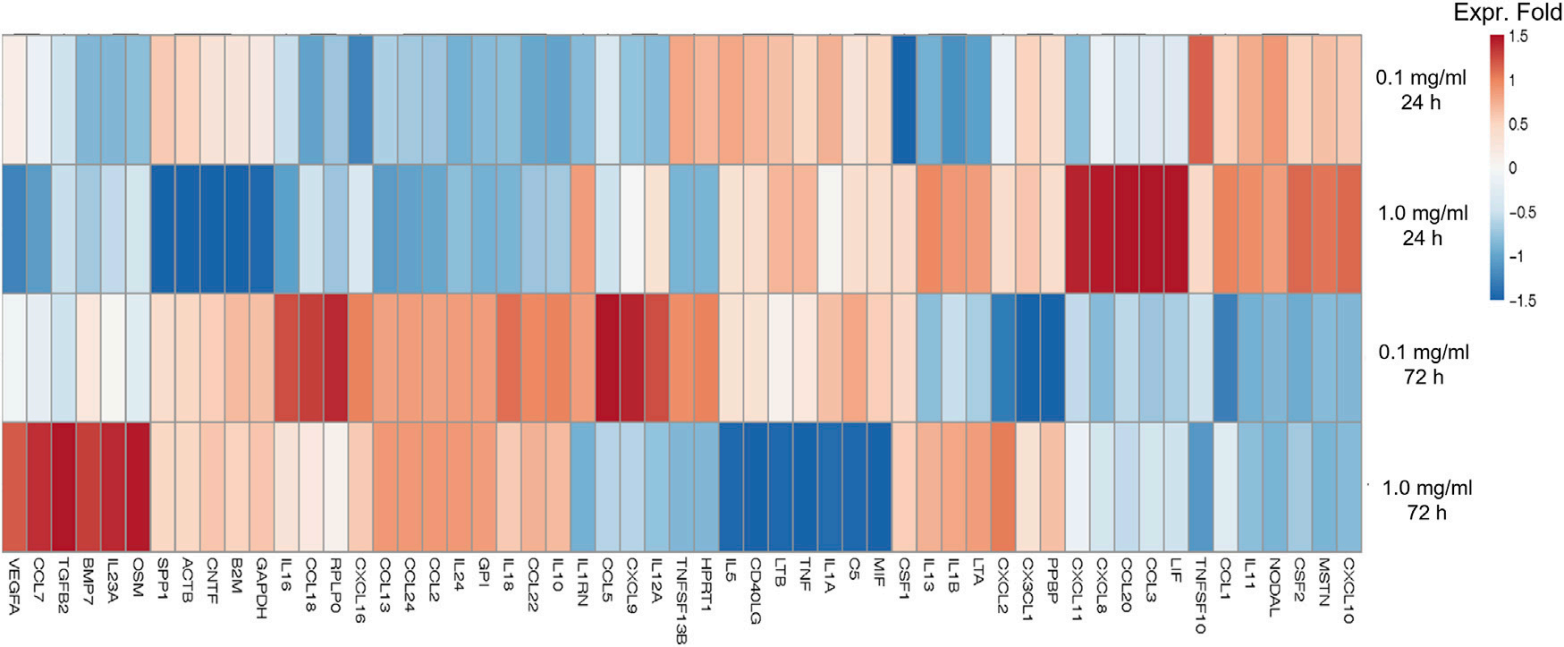
Heatmap of genes associated with inflammation of macrophages treated with low molecular weight of sericin solution for 24 and 72 h. This heatmap was built using DESeq 2 on normalized gene read counts. All the log values of the dispersion estimates were clustered using the R distance function (dist) to calculate the Euclidean distance between samples. Distance plot showing 24 and 72 h treatment with sericin-treated versions. Red: upregulated gene; blue: downregulated gene.

We assessed the effect of LMW-sericin treatment on macrophage viability using the MTT assay for 1 and 3 days of incubation. The results showed that the concentration of the LMW-sericin solution affected cell viability; it was significantly decreased upon the increase of solution concentration and incubation time compared to the control, with mean viable cell percent ±SE values of 100.00 ± 9.24, 120.40 ± 11.69, 114.51 ± 4.72, 81.73 ± 17.70, and 26.39 ± 3.32 for 0, 0.1, 0.5, and 1 mg/ml concentrations and 10% DMSO, respectively, in 24 h, and 100.00 ± 28.54, 144.18 ± 37.16, and 60.72 ± 34.53, 65.26 ± 40.67, and 10.02 ± 1.01 for 0, 0.1, 0.5, and 1 mg/ml concentrations and 10% DMSO, respectively, in 72 h (Figure 3). We further observed the regulation of gene expression associated with inflammation in macrophages treated with LMW-sericin and demonstrated its alteration using heatmap analysis. After 1 day of incubation, macrophages cultured with 0.1 mg/ml concentration of LMW-sericin displayed upregulation of IL-5 and TNF superfamily members including LTB and TNFSF10, while macrophages cultured with 1 mg/ml LMW-sericin displayed upregulation of inflammatory cytokines and chemokines including CXCL9, IL-1β, and CXCL8; growth factors including LIF, MSTN, and CSF2; TNF superfamily members including LTB and TNFSF10 (Figure 4). After 3 days of incubation, macrophages cultured with 0.1 mg/ml concentration of LMW-sericin displayed upregulation of immune response cytokines including CXCL9, IL12A, and BMP7 as well as anti-inflammatory cytokine IL10, while macrophages cultured with 1 mg/ml LMW-sericin displayed upregulation of IL-1β, growth factors including BMP7 and OSM as well as anti-inflammatory cytokine TGFB2. These results showed the sericin concentration from 1 to 10 times to evaluate the macrophage proliferation in a dose-dependent manner (Figure 3) and the modulation of immunity by macrophages through the expression of inflammatory cytokines and chemokines, growth factors, and anti-inflammatory cytokines (Figure 4).

**FIGURE 5:**
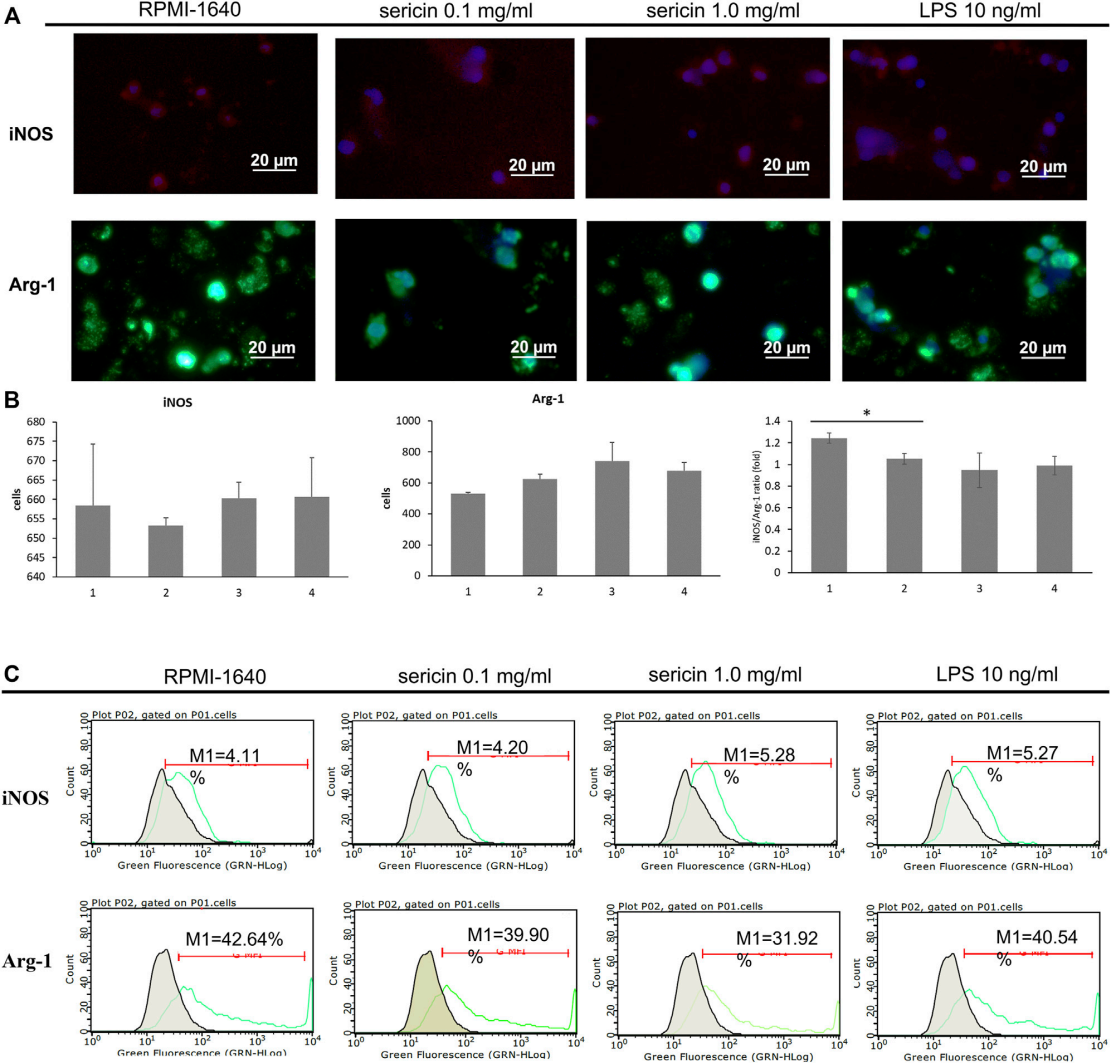
Macrophage polarization in response to LMW-sericin treatment after 24 h. **(A)** Immunofluorescence staining images of macrophages stained for DAPI (blue), M1 surface marker iNOS (red), and M2 surface marker Arg-1 (green); **(B)** Semi-quantitative analysis of macrophages expression by each marker. This analysis was automatically performed by ZEN 2.6 lite blue software; **(C)** Flow cytometry analysis of M1/M2 macrophages. (1 = PMA + RPMI-1640; 2 = PMA + sericin 0.1 mg/ml; 3 = PMA + sericin 1.0 mg/ml; 4 = PMA + LPS 10 ng/ml; scale bar = 20 μm; *n* = 6 per group per condition, six random fields per well; *p < 0.05).

During inflammation, the induction of macrophage polarization is closely linked to the local environment that acts as a pathogens’ destroyer or repairs the tissues and maintains the homeostasis. According to cell viability result (Figure 3), sericin had a 35%–40% cell mortality rate at 72 h of treatment, thus, we used 24 h of treatment time to observe the differentiation of macrophages in the following experiment. In this study, the effect of LMW-sericin treatment on macrophage polarization was observed by employing M1-related marker iNOS (red) and M2-related marker Arg-1 (green) for immunofluorescence staining. As shown in Figure 5A, the expression of Arg-1 M2 marker was noticed more intensely within all groups than the expression of iNOS M1 marker. The quantitative analysis showed no significant difference between each group for iNOS M1 marker, where the mean of positive cells ±SE values was 658.39 ± 5.82, 653.19 ± 2.07, 660.29 ± 4.12, and 660.60 ± 10.13 for PMA + RPMI-1640, PMA + sericin 0.1 mg/ml, PMA + sericin 1.0 mg/ml, and PMA + LPS 10 ng/ml, respectively (Figure 5B). Furthermore, both LMW-sericin treatment groups demonstrated a higher level of Arg-1 M2 marker compared to the control, where the mean of positive cells ±SE values was 529.90 ± 9.31, 624.26 ± 30.34, 739.06 ± 122.34, and 677.23 ± 53.45 for PMA + RPMI-1640, PMA + sericin 0.1 mg/ml, PMA + sericin 1.0 mg/ml, and PMA + LPS 10 ng/ml, respectively. Furthermore, this quantitative analysis was used to calculate the iNOS/Arg-1 ratio, while the ratio was used to determine the polarization dominance of M1 and M2. The regulation of iNOS/Arg-1 ratio was significantly decreased in 0.1 mg/ml concentration of LMW-sericin treatment compared to the control (*p* < 0.05), indicating the dominance of Arg-1. In addition, macrophages after polarization were analyzed by using flow cytometry (Figure 5C), and the results showed the cell counts of M1 and M2 markers. Contrary to what was expected, the levels of all markers were less variable impeding the assignment of a clear M1 or M2 phenotype. Also, the basal expression levels were almost non-variable between groups, despite the highly similar differentiation stage. For Instance, PMA + LPS group expresses more of the M1 marker percentage than LMW-sericin treatment groups, as well as M2 marker. Interestingly, even the basal levels of expression were less variable within sericin treatment, suggesting insufficient responsive cellular activation. In agreement with that, PMA treatment alone often resulted in the level of M1 or M2 marker similar to after M1- or M2-polarizing condition.

### 3.4 The Effects of Low Molecular Weight Sericin Treatment on Human Adipose Stem Cell Behavior

**FIGURE 6:**
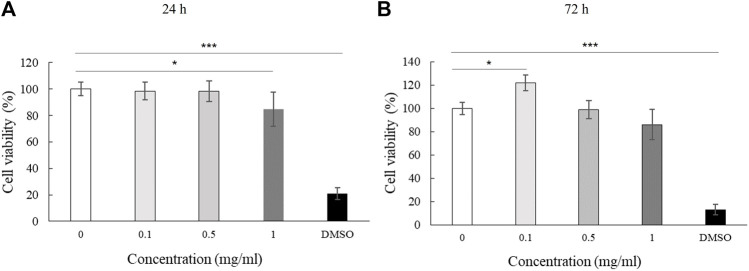
Cell viability of human adipose stem cells (hASCs) cultured with low molecular weight of sericin solution. (*n* = 3; *p < 0.05, ***p < 0.001).

Stem cells are involved in all overlapping phases of wound repair and could release various chemo-cytokines and growth factors to stimulate the healing process; hence, the evaluation of stem cells' behavior towards wound treatment is substantial. We first investigated the effect of LMW-sericin treatment on hASCs viability using the MTT assay for 1 and 3 days of incubation. The results demonstrated the mean viable cell percent ±SE values of 100.00 ± 5.17, 98.54 ± 6.62, 98.20 ± 7.84, 84.86 ± 12.97, and 20.82 ± 4.39 for 0, 0.1, 0.5, and 1 mg/ml concentrations and 10% DMSO, respectively in 24 h, and 99.99 ± 13.70, 121.94 ± 11.83, and 98.76 ± 20.29, 85.97 ± 22.76, 13.00 ± 3.46 for 0, 0.1, 0.5, and 1 mg/ml concentrations and 10% DMSO, respectively in 72 h (Figure 6), showing that the percentage of hASC viability was significantly increased at a concentration of 0.1 mg/ml after 72 h of incubation compared to the control, with no substantial effect observed after 24 h of incubation.

**FIGURE 7:**
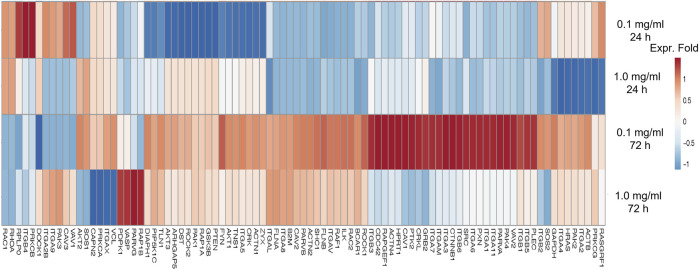
Heatmap of genes associated with adhesion molecule of human adipose stem cells treated with low molecular weight of sericin solution for 24 and 72 h. This heatmap was built using DESeq 2 on normalized gene read counts. All the log values of the dispersion estimates were clustered using the R distance function (dist) to calculate the Euclidean distance between samples. Distance plot showing 24 and 72 h treatment with sericin-treated versions. Red: upregulated gene; blue: downregulated gene.

Furthermore, the regulation of gene expression associated with adhesion molecules in hASCs treated with LMW-sericin was observed and demonstrated using heatmap analysis (Figure 7). After 1 day of incubation, hASCs cultured with 0.1 mg/ml concentration of LMW-sericin demonstrated upregulation of genes related to integrin, focal adhesion, G protein signaling, caveolin activation, and AKT/PI-3-kinase pathway, respectively, including ITGA2B, PRKCB, RASGRF1, VAV1, and CAV3, while hASCs cultured with 1 mg/ml LMW-sericin demonstrated the downregulation of those genes. After 3 days of incubation, all the associated genes were upregulated with 0.1 mg/ml concentration of LMW-sericin treatment including ITGA1, ITGA2, ITGA4, ITGA3, ITGA5, ITGA7, ITGA11, ITGAL, ITGB1, ITGB2, ITGB5, SRC, GRB2, SHC1, ACTN1, ACTN2, AKT1, FLNA, FLNB, TLN1, VASP, ZYX, ILK, PARVA, PARVB, CRK, CRKL, FYN, PAK4, RAF1, RASGRF1, ROCK1, VAV2, RAC2, CTNNB1, PIP5K1C, PXN, TNS1, VASP, ZYX, PLEC, and DIAPH1, associated with cell growth and differentiation, cell survival, cell migration, and cell elongation, through the activation of signaling pathways such as the MAPK, PI3K, and Wnt signaling pathways, and the regulation of actin cytoskeleton, respectively, while the treatment with 1 mg/ml concentration of LMW-sericin displayed this tendency less.

**FIGURE 8:**
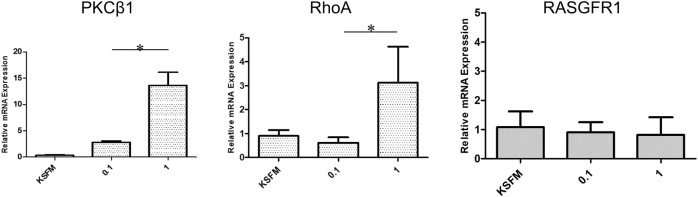
mRNA expression of protein kinase enzymes including PKCβ1, RhoA, and RasGFR1 generated by human adipose stem cells (hASCs) cultured with low molecular weight of sericin solution for 72 h (*p < 0.05).

**FIGURE 9:**
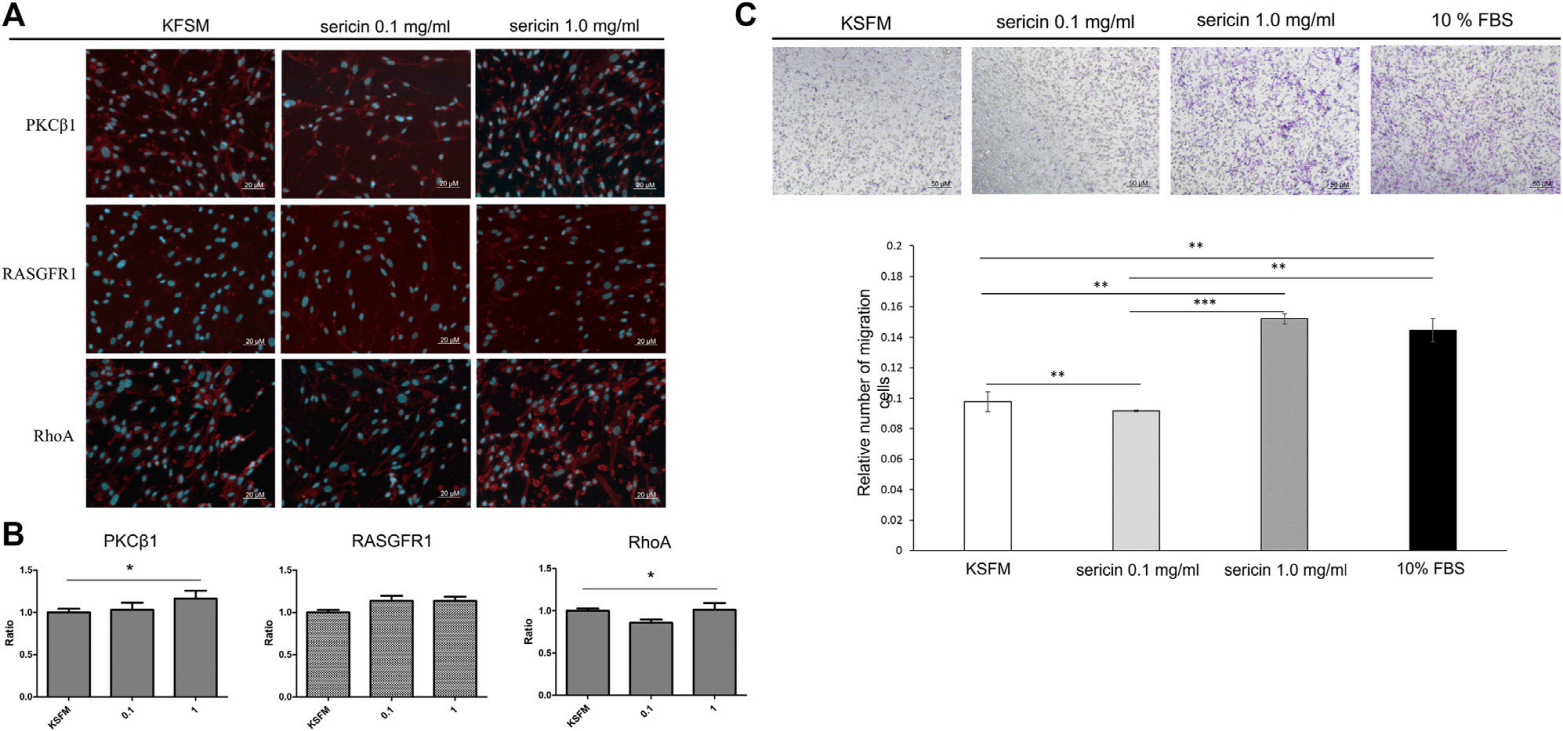
Human adipose stem cells (hASCs) in response to low molecular weight of sericin solution treatment after 72 h. **(A)** Immunocytochemistry staining images of hASCs stained for DAPI and PKCβ1, RhoA, and RasGFR1 markers, respectively (scale bar = 20 μm); **(B)** quantitative analysis of positive hASC expression by each marker; **(C)** cell migration assay (scale bar = 50 μm). (*p < 0.05, **p < 0.01, and ***p < 0.001; *n* = 6 per group per condition, six random fields per well).

During the healing process, wound closure is a well-coordinated biological process, requiring directed cell migration toward the center of the wound. According to the qRT-PCR results (Figure 7), LMW-sericin potentially improved hASCs cell replication and migration, and the expression of PKC protein family factors was upregulated. Thus, we further focused on the evaluation of protein kinase enzymes including PKCβ1, RhoA, and RasGFR1 as fruitful molecules in wound healing. In our study, PKCβ1, RhoA, and RasGFR1 transcript levels were detected, suggesting that these genes were active at basal levels in the presence of stem cell migration conditions and confirmed their potential as master activators and regulators of cell motility. The expression of genes where the ratio of fold change ± SE values was detected in KSFM, 0.1, and 1 mg/ml groups, respectively. PKCβ1: 0.32 ± 0.11, 2.81 ± 0.27, and 13.6 ± 2.52; RasGFR1: 1.07 ± 0.54, 0.90 ± 0.34, and 0.81 ± 0.60; RhoA: 0.90 ± 0.24, 0.62 ± 0.22, and 3.11 ± 1.50 (Figure 8). Although our results show that PKCβ1 and RhoA expression describes an ascendant trend post sericin treatment (Figure 8), statistically significant increases in gene expression were registered at LMW-sericin 1.0 mg/ml concentration (*p* < 0.05) when compared to LMW-sericin 0.1 mg/ml concentration, while the expression of RasGFR showed no significant differences. In the intracellular expression of PKCβ1, RhoA, and RasGFR1, the immunocytochemistry staining images showed that hASCs-treated LMW-sericin demonstrated denser cells attached compared to the untreated control group (Figure 9A). The total number of double DAPI and marker-positive cells in the hASCs in each field was also counted, where the ratio of positive cells ± SE values was detected in KSFM, 0.1, and 1 mg/ml groups, respectively. The semi-quantification value was 1.00 ± 0.04, 1.03 ± 0.08, and 1.16 ± 0.09; for PKCβ1; 1.00 ± 0.03, 1.14 ± 0.06, and 1.14 ± 0.05 for RasGFR1; and 1.00 ± 0.03, 0.86 ± 0.04, and 1.01 ± 0.08 for RhoA (Figure 9B). We detected a significant upregulated PKCβ1 and RhoA (*p* < 0.05) in the presence of sericin at 1.0 mg/ml as compared to KSFMs, while the expression of RasGFR displayed no significant differences although showing an increasing trend with LMW-sericin treatment. Moreover, the results of cell migration assay demonstrated that the OD proportion of hASCs, which represented the relative number of migration cells, was 93.85% (0.1 mg/ml concentration) and increased by 55.83% (1.0 mg/ml concentration) compared with the control group (*p* < 0.01; Figure 9C). In addition, hASCs treated with LMW-sericin 1.0 mg/ml were increased by 66.03% compared with the sericin 0.1 mg/ml treatment (*p* < 0.001). The results exhibited that the number of PKCβ1-, RasGFR1-, and RhoA-positive cells in the hASCs was meaningfully influenced by LMW-sericin treatment in the control group, implying that LMW-sericin promotes the proliferation and migration of hASCs through the PKC signaling pathway.

## 4 Discussion

Sericin has been reported to possess various biological functions that are beneficial for biomedical applications, including cancer prevention, wound healing, and drug delivery (Zhang, 2002; Kaewkorn et al., 2012; Aramwit et al., 2013). As the bioactivities of sericin are significantly influenced by its MW, in this study, we extracted LMW-sericin by the HTHP method and evaluate its effects on the immunomodulation and potential signaling pathways of cell behavior in wound healing. Firstly, we confirmed the MW of the LMW-sericin solution obtained by this degumming condition matched the proposed strategy, which was less than 10 kDa, by performing MALDI-TOF and LC-MS (Figure 1). This might be because of the HTHP environment strongly cleaved the sericin protein chain, resulting in MW reduction during the silk degumming process. Hence, this range of MW is expected not only to avoid the possibility of inducing an allergic reaction but also to optimize the absorption and provide nutrient substrates for wound healing (Yang et al., 2018). Degumming under high temperature and pressure conditions, unlike chemical degumming, is an economic producing technique with no toxic and harmful effect on the fiber or environment. As far as environmental welfare is concerned, the consumption of chemical methods carries serious pollution to the land and water systems. To obtain good yields of sericin of the desired MW and to avoid any chemical contamination, therefore, the heating conditions were chosen as the silk degumming method. Lamoolphak et al. (2008) reported the hydrothermal degumming in sealed reactors and investigated the effects of different temperatures, reaction times, and ratios of silk to water on sericin protein and amino acid production. The authors found optimal sericin yields at 130°C and 60 min of degumming process; this finding was also presented by another study, which indicated that the autoclave degumming has produced an excellent quality of muga silk in terms of sericin loss, surface smoothness, and fiber strength in contrast to conventional degumming (Choudhury and Devia, 2016). Based on their results, the same degumming procedure was thus used as a suitable condition in this study.

The molecular conformation of the LMW-sericin protein was also examined *via* FT-IR spectroscopy. The protein molecules presented such characteristics of energy absorption between wave numbers 1,700 and 1,600 cm^−1^ for amide I, between wave numbers 1,575 and 1,480 cm^−1^ for amide II, and between wave numbers 1,330 and 1,230 cm^−1^ for amide III, and approximately 3,400 cm^−1^ for amide A (Garidel and Schott, 2006). The peak positions of amides A, I, II, and III correspond to C=O stretching vibration, N-H bending and C-N stretching vibrations, and C-N stretching vibration coupled to the N-H in-plane bending vibration, respectively (Krimm and Bandekar, 1986). Additionally, amide A absorption primarily represents the stretching vibrations of the N-H groups. The IR absorption peaks of silk fibroin (0% sericin) were observed at 1,620 and 1,510 cm^−1^, which were attributed to the β-sheet crystalline silk (Jo and Um, 2015; Jang and Um, 2017). Our results showed that amide I of LMW-sericin was shifted to 1,637 cm^−1^ (Figure 2A), confirming that the heat treatment transformed the β-sheet crystalline into a random coil, as a result of increased sericin content. Furthermore, the proportions of the β-sheet crystalline and random coil conformations of the LMW-sericin were evaluated by deconvoluting the amide I bands (Figure 2B). The absorptions of amide I (the region around 1,700–1,600 cm^−1^) can be attributed to the differences in protein secondary structure due to the sensitivity of the hydrogen bonding interaction (Teramoto and Miyazawa, 2005). The proportion of each secondary structure of sericin film based on the peak areas of the component bands was 27.5% for aggregated strands, 26.5% for β-sheets, 40.9% for random coils, and 5.1% for α-helices (Table 1). This result proved that random coil is the most abundant structural component of LMW-sericin extracted by the HTHP degumming method without any significant changes in the secondary structure, in line with previous research (Teramoto and Miyazawa, 2005; Zhang and Wyeth, 2010).

**FIGURE 10:**
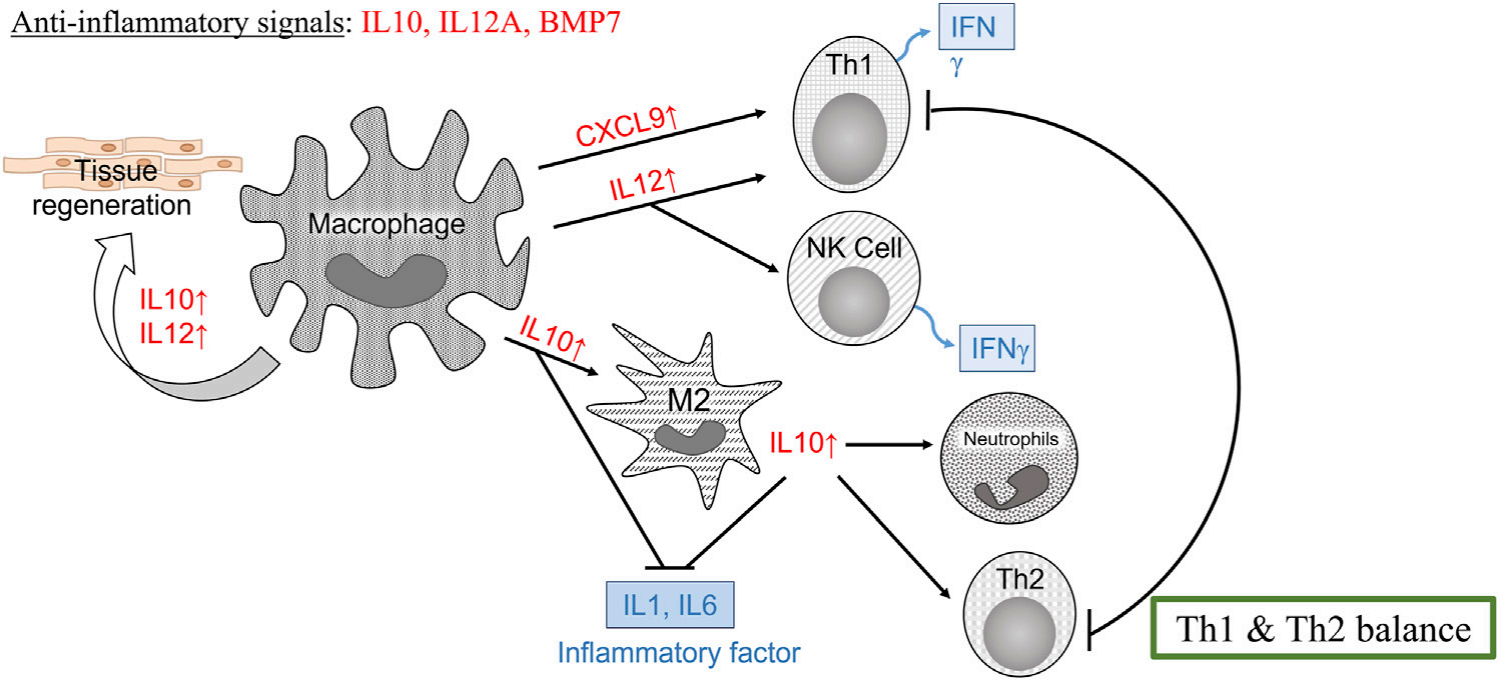
Schematic illustration of the immune response according to the gene expression of macrophages cultured with 0.1 mg/ml low molecular weight of sericin solution for a 3-day incubation.

For biomedical applications, the biocompatibility of materials is an essential parameter that must be considered. Hence, we examined the effect of LMW-sericin treatment on the viability of macrophages and hASCs after 1 and 3 days of incubation (Figures 3, 6). Although the results exhibited different consequences for each cell type, LMW-sericin at a concentration of 0.1 mg/ml showed the greatest cell viability. The result indicated that LMW-sericin demonstrated good biocompatibility with the cell environment, with a percentage of cell viability of more than 80%. We further evaluated the effects of LMW-sericin on macrophage behavior through gene expression to estimate its potential immune response. After 3 days of incubation with LMW-sericin at a concentration of 0.1 mg/ml, macrophages demonstrated an improved inflammatory response (Figures 4, 10). Together with IL12 that stimulates the generation of Th1 and NK cells for initiating host defense and inflammation (Song et al., 2020), the upregulated IL10 acts as an anti-inflammatory agent responsive to the Th2-producing cells for maintaining the immune homeostasis in mucosal tissues and facilitating tissue repair (Wynn and Vannella, 2016). This circumstance develops Th1 and Th2 balance, which is beneficial to the balance of inflammation, regeneration, and remodeling to restore the damaged tissue (van Eden et al., 2002; Boothby et al., 2020). Additionally, there was an upregulation in CXCL9 and BMP7 expression, which are also involved in the balance of Th1 and Th2 responses (Figure 10). During this dynamic homeostasis, the relative suppression of Th1 cells by the relative increase in Th2 activity is the key mechanism for maintaining or restoring balance in a diseased immune system (Ross et al., 2021). These results indicate that LMW-sericin tends to induce M2 activation in macrophages, along with a classic activation of M1 macrophages. This outcome is further confirmed by the analysis of macrophage polarization after LMW-sericin treatment (Figure 5). LMW-sericin treatment, especially in 0.1 mg/ml concentration, remarkably increased the expression of Arg-1 M2 marker. Studies have demonstrated the key roles of M2 pathways in mediating the feature of the self-promoting process in macrophage function by providing an important survival pathway that eliminates the damaged cells *via* efferocytosis and guide the resolution of inflammation and tissue repair pathways (Korns et al., 2011; Ross et al., 2021). Overall, these results demonstrated that LMW-sericin might induce the differentiation of macrophages towards the M2 phenotype, which is conducive to the repair of the inflammatory environment.

In therapeutic reactions, the mechanism of inflammation also involves the presence of stem cells that can differentiate into specific cells and transmit various signals for the assembly of different cell types required for tissue repair. The balance between the inflammatory response and its mediated stemness can help to maintain tissue integrity and promote aberrant homeostasis and disease (Michael et al., 2016). Therefore, it is important to examine the bio-responses of stem cells as a result of this signaling to evaluate the effectiveness of the treatment. In this study, we used hASCs, potential adult stem cells based on their ability to readily generate both ectodermal and mesodermal cell lineages, to provide miscellaneous strategies for clinical applications (Cherng et al., 2014). The cell viability test results showed that the percentage of hASC viability compared to without LMW-sericin treatment was significantly increased at a concentration of 0.1 mg/ml after 3 days of incubation (Figure 6), indicating that this treatment provides a suitable environment for hASCs to proliferate well. Moreover, the effects of LMW-sericin on hASCs were evaluated through gene expression to estimate the potential cell signaling pathways. After 3 days of incubation, 0.1 mg/ml LMW-sericin treatment of hASCs upregulated various genes associated with adhesion molecules and implied some possible signaling pathways related to cell behaviors (Figure 7). RAS is required to recruit RAF1; on the other hand, RAF1 activation was also strongly supported by the phosphorylation of PAK through the phosphatidylinositol signaling system (Wary et al., 1998). These sequences may activate the RAF-MEK-ERK (MAPK) signaling pathway and delineate cell growth and differentiation to cellular adaptation to chemical and physical stress under a wound environment (Chaudhary et al., 2000; Chang and Karin, 2001). Furthermore, we found that both concentrations of LMW-sericin enhanced the expression of protein kinase enzymes including PKC, RhoA, and RasGFR1, suggesting the improvement of hASCs replication and migration (Figures 8, 9). These results were slightly different from the gene expression results where only 0.1 mg/ml LMW-sericin treatment demonstrated its significant influence on the genes-related PKC signaling pathway. It might be due to the effects of post-translational modifications on protein stability; hence, mRNA and protein expression levels in cell-culture systems may not always be correlated. Nevertheless, it is still implied that LMW-sericin could better influence the stem cell behavior for wound healing when compared to the untreated stem cells. The phosphorylation of PKC induced by LMW-sericin is essential for cellular migration because PKC activation is important in cytoskeletal rearrangement and cell behavior including cell proliferation, survival, and death (Reyland, 2009; Chae et al., 2018). We suggest that priming stem cells with LMW-sericin may therefore improve the initial interaction between transplanted stem cells and host tissue cells, resulting in enhanced therapeutic efficacy. In addition, Wang et al. (2017) have proposed that RhoA-mediated cyclin D1 upregulation *via* PKN1 activation is a new mechanism for stem cell proliferation, implying that RhoA may play a role in wound healing. RhoA has been demonstrated to majorly manage actin-based structure formation, cell proliferation, and cell migration (Zhang et al., 2015; Hu et al., 2016; Tseliou et al., 2016). Our data show that RhoA is a downstream mediator of sericin-induced migration in hASCs. Furthermore, PKCβ1 and RasGFR1 are signaling key events of sericin-induced cell migration and their actions occur through the downstream mechanisms of RhoA activation.

One of the novelties of this study is that we provide low-cost and environmentally friendly methods for sericin isolation. Our results show the utilization of eco-friendly and less hazardous methods of autoclave degumming for the production of low molecular weight sericin with low immunity and non-toxicity to cells, providing new horizons for regenerative medicine. The *in vivo* wound healing studies and their mechanism behind them are needed to be investigated. Furthermore, as sericin has been recognized to have immunomodulatory activities, further investigations are warranted to evaluate sericin as an immunomodulator under inflammatory responses, such as sericin regulating inflammatory responses in allergy or autoimmune disease of animal models. Overall, based on all these signaling pathways, LMW-sericin stimulates the secretion of beneficial adhesion molecules from hASCs, which may activate the gene transcription associated with differentiation and migration in hASCs, which can regulate and regenerate inflamed tissues. Findings of this study will expand the progress of current tissue engineering by providing insights into stem cell therapy for wound healing.

## 5 Conclusion

This study demonstrated that LMW-sericin not only displayed good biocompatibility but also positively regulated the immune response in macrophages and behavior for wound healing in stem cells. LMW-sericin showed *in vitro* anti-inflammatory activity in macrophages as well as induced their polarization towards the M2 phenotype, which is conducive to the restoration of the inflammatory environment and encouraging tissue repair. Moreover, LMW-sericin may stimulate the secretion of beneficial adhesion molecules from hASCs, which in turn activate the gene transcription associated with differentiation and migration in hASCs, thereby regulating and regenerating inflamed tissues. These findings provide insights into LMW-sericin application as a potential biological agent for accelerating wound healing.

## Data Availability

The original contributions presented in the study are included in the article/Supplementary Material; further inquiries can be directed to the corresponding author.
